# Improvement in Luminescence Intensity of β-NaYF_4_: 18%Yb^3+^, 2%Er^3+^@β-NaYF_4_ Nanoparticles as a Result of Synthesis in the Presence of Stearic Acid

**DOI:** 10.3390/nano12030319

**Published:** 2022-01-19

**Authors:** Piotr Kaminski, Dominika Przybylska, Gabriela Klima, Tomasz Grzyb

**Affiliations:** Department of Rare Earths, Faculty of Chemistry, Adam Mickiewicz University in Poznań, Uniwersytetu Poznańskiego 8, 61-614 Poznań, Poland; piotr.kaminski@amu.edu.pl (P.K.); dominika.przybylska@amu.edu.pl (D.P.); gabkli1@st.amu.edu.pl (G.K.)

**Keywords:** nanoparticles, synthesis, stearic acid, core@shell, lanthanides, upconversion

## Abstract

The synthesis of upconverting nanoparticles (NPs) is crucial for their spectroscopic properties and further applications. Reducing the size of materials to nano-dimensions usually decreases emission intensity. Therefore, scientists around the world are trying to improve the methods of obtaining NPs to approach levels of emission intensity similar to their bulk counterparts. In this article, the effects of stearic acid on the synthesis of core@shell β-NaYF_4_: 18%Yb^3+^, 2%Er^3+^@β-NaYF_4_ upconverting NPs were thoroughly investigated and presented. Using a mixture of stearic acid (SA) with oleic acid and 1-octadecene as components of the reaction medium leads to the obtaining of monodispersed NPs with enhanced emission intensity when irradiated with 975 nm laser wavelength, as compared with NPs prepared analogously but without SA. This article also reports how the addition of SA influences the structural properties of core@shell NPs and reaction time. The presence of SA in the reaction medium accelerates the growth of NPs in comparison with the analogic reaction but without SA. In addition, transmission electron microscopy studies reveal an additional effect of the presence of SA on the surface of NPs, which is to cause their self-organization due to steric effects.

## 1. Introduction

Recently, upconverting nanoparticles (UCNPs) have become one of the most important topics in materials sciences. The reasons for the ever-growing interest in UCNPs include their spectroscopic properties and their numerous related applications. The UCNPs are capable of a multi-photon excitation under near-infrared (NIR) light radiation followed by the emission of radiation with a higher-than-absorbed energy, usually in the range of ultraviolet to visible light [1,2]. The spectroscopic properties of UCNPs are determined by the unique properties of lanthanide ions (Ln^3+^) and their electronic transitions within the inner 4f shell. As a result of the shielding effect by outer 5s and 5p shells, the lifetimes of the excited 4f states are relatively long, which allows for the upconversion process to occur [3].

The upconverting particles can be considered excellent alternatives to semiconductor quantum dots or organic dyes because their excitation does not cause biological autofluorescence. UCNPs present good light stability, low material toxicity, and their properties allow for deeper penetration of excitation light than is the case for ultraviolet or visible radiation [4,5,6,7]. UCNPs are also characterized by a high shift between the absorption and emission bands, which is especially demanded by bioimaging techniques [4,5,6,7]. These unique properties make the UCNPs very interesting and widely applied in many fields: in electronic devices such as three-dimensional displays; to protect documents or analytical and biomedical applications (e.g., immunoassays, bioimaging, drug delivery, photodynamic, and photothermal therapies) [8,9,10,11,12].

The properties and applications of nanoparticles (NPs) are strongly related to the methods of their synthesis. The main reason UCNPs are susceptible to the type of synthesis method is because of the sensitivity of the upconversion process to structure defects, the presence of water, and quenching processes caused by a relatively large ratio of NPs’ surface area to volume. So far, it has been proven that inorganic fluorides are perfect matrices for Ln^3+^ ions showing the upconversion phenomenon and that covering UCNPs with an additional shell (in core@shell structures) minimizes quenching caused by surface factors. Nowadays, optimizing the UCNPs’ synthesis is under intensive study [13].

In most reports concerning UCNPs, sodium-yttrium fluoride (NaYF_4_) is used as a host for Ln^3+^ ions. Low phonon energy and good stability of this compound allow a high efficiency of the emission of materials based on it to be obtained [13]. Here, NaYF_4_ fluoride was also used, as a model structure, allowing the evaluation of the obtained effects in the context of other publications. Depending on the synthesis conditions, the NaYF4 crystals can be obtained in two structures: the cubic (α) and the hexagonal phase (*β*). UCNPs based on the hexagonal NaYF_4_ can generate a much higher emission intensity than the cubic phase [14].

NaYF_4_-based materials are usually synthesized by hydrothermal and solvothermal methods or by the reaction of high-boiling point solvents. The latter one is the most important for the synthesis of core@shell UCNPs and also the most frequently used. In this type of synthesis, the most common solvents are oleic acid (OA), 1-octadecene (ODE), and oleylamine (OM) [13,15,16]. As the NP growth mechanism of the NPs’ growth is based on the Ostwald ripening, a modification of the medium in which the whole reaction takes place may influence the properties of the final products [17,18]. For example, the addition of OM as a surface capping ligand allows for the growth of nanoparticles to be controlled by changing the conversion mechanism of the cubic to the hexagonal phase [16]. The increase in the ODE fraction in the composition of the mixture of organic solvents can affect the shape of core@shell β-NaYF_4_: 18%Yb^3+^, 2%Er^3+^ @β-NaYF_4_ materials by giving the product in the form of nanorods [19]. A similar effect was obtained by adding trioctylphosphine to the reaction medium [20].

Stearic acid (SA) as a component of the reaction medium and, next, ligand adsorbed to the surface of NPs may also dictate their nucleation and growth processes. Examples of the use of the properties of SA in the synthesis of NPs are not many and include the synthesis of CdSe QDs [21], rare earth oxyfluorides [22], and metallic silver NPs [23]. However, one of the most important effects that SA, playing the role of ligands, can introduce is the lowering of surface quenching by replacing OA molecules. It was recently demonstrated that the capping ligands’ conformation significantly influences the luminescence quenching from both ligand and solvent molecules and can be a major reason for the size-dependent luminescence quenching of the UCNPs [24].

The search for a way to improve the luminescence properties of UCNPs and their synthesis procedure was one of the main motives of the research presented here. Adding SA to the reaction medium is an easy step as it does not significantly change the known methods of core@shell UCNPs synthesis. However, we prove that this minor modification is of great importance. To the best of our knowledge, no previous attempts to use SA to synthesize core@shell NPs have been reported.

## 2. Experimental Part

### 2.1. Materials

All reagents were used as obtained without further purification. The rare earth oxides Er_2_O_3_ (99.99%), Y_2_O_3_ (99.99%), and Yb_2_O_3_ (99.99%) were purchased from Stanford Materials (Lake Forest, CA, USA). Hydrochloric acid (35–38%) was purchased from the Avantor Performance Materials (Gliwice, Poland), ethanol (99.8%) was purchased from Wyborowa S.A. (Poznań, Poland), and n-hexane (≥99%) was purchased from Honeywell Poland (Warszawa, Poland). The solvents, 1-octadecene (1-ODE, 90%), and oleic acid (OA, 90%), were purchased from Alfa Aesar (Haverthill, MA, USA). Sodium oleate (82%) was bought and stearic acid (SA, 95%) was obtained from Sigma Aldrich (Saint Louis, MO, USA). Aqueous solutions of hydrochloric acid with rare earth oxides were prepared by dissolution in demineralized water. Fluoride syntheses of 99.99% purity were performed under nitrogen flow from Linde (Poznań, Poland).

### 2.2. Synthesis of Materials

#### 2.2.1. Synthesis of Rare Earth Precursors

Before the syntheses, the precursors of rare earth materials were obtained. For the synthesis of each 2 mmol of rare earth chloride (erbium, ytterbium, or yttrium chloride), 1 mmol of rare earth oxide was dissolved in 25 mL of demineralized water and 5 mL of hydrochloric acid. The rare earth oxide in aqua hydrochloric acid solution was heated to 70 °C and kept at this temperature overnight (for 16 h) at the stirring speed of 300 rpm and under a reflux condenser. Then, a clear solution was evaporated using a rotary evaporator (Heidolph) under reduced pressure (of around 100 mbar) for approximately 2 h. The final chlorides were obtained after drying at 110 °C overnight under atmospheric pressure.

#### 2.2.2. Synthesis of Nanomaterials

**Scheme 1:**
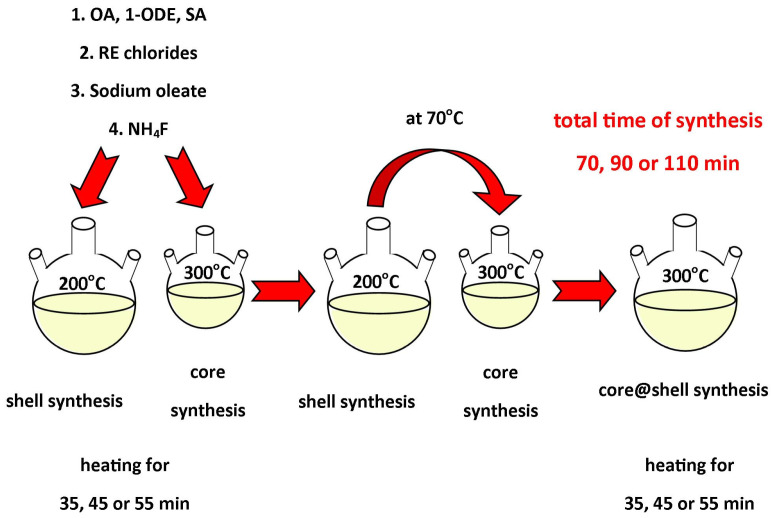
The procedure of synthesis of NPs in a mixture of oleic acid (OA), 1-octadecne (1-ODE), and stearic acid (SA).

In this work, all of the syntheses of NPs were performed in the mixture of OA, 1-ODE, and SA. In the β-core and α-shell NPs syntheses, the molar ratios of the ions Na^+^:Ln^3+^:F^−^ were 3:1:6 and 3:2:8, respectively. In all syntheses of C1-C3 and CS1-CS3 NPs, OA, 1-ODE, and SA were used at the ratio of 1 mmol (α-shell or β-core): 5 mL:10 mL:5 mL. In the first step, β-core and α-shell NPs were prepared in the parallel syntheses. The main difference between the syntheses of both groups of materials was the temperature. The α-shell NPs were prepared at 200 °C and β-core materials at 300 °C. The solutions with α-shell and β-core NPs were used in the second step—the synthesis of core@shell materials at 300 °C. The list of all obtained and characterized materials is presented in Table 1, while Scheme 1 shows the procedure of core@shell NPs synthesis.

#### 2.2.3. Synthesis of β-core NaYF_4_:18%Yb^3+^, 2%Er^3+^ and α-shell NaYF_4_ NPs

At the beginning, the organic solutions OA, 1-ODE, and SA were poured into two three-neck flasks connected to a reflux condenser and a Schlenk line (one flask for the synthesis of β-core NPs and another one for the synthesis of α-shell NPs). Then, the mixture of solutions was heated from room temperature to 70 °C under the nitrogen flow and, when the stearic acid was dissolved, the heating of the mixture was continued to 100 °C under low pressure (<10^−1^ mbar). The solution was outgassed at this temperature for 60 min. Following this, the powders of rare earth chlorides (yttrium, ytterbium, and erbium in the case of β-core NaYF_4_:18%Yb^3+^, 2%Er^3+^ materials and yttrium chloride in the case of α-shell NaYF_4_) were added to the solution under the flow of nitrogen at 100 °C. The mixture was heated at 100 °C under low pressure (<10^−1^ mbar) for 60 min. Then, sodium oleate as a powder was added to the solution at 100 °C in nitrogen flow. After outgassing the mixture for 20 min, the source of sodium ions was dissolved, and the solid ammonium fluoride was added to the solution at 100 °C under the flow of nitrogen. Then, the solution was heated to 300 °C (in the case of β-core NPs synthesis) or 200 °C (in the case of α-shell NPs synthesis). The solution was kept at a selected temperature for 35 (C1 sample), 45 (C2 sample), or 55 min (C3 sample) and then cooled to 70 °C under the flow of nitrogen. Upon cooling the solution, pure ethanol was heated from room temperature to 70 °C under the nitrogen flow. This warm ethanol was used to precipitate NPs from the mixture of organic solutions because stearic acid dissolves very well in warm pure ethanol. Before separating NPs, the solution with NPs (whose temperature was around 70 °C) was poured into the centrifuge probes with pure warm ethanol and centrifuged (at 9000 rpm for 3 min). After the centrifugation, the solution was removed, and the precipitate of NPs was dissolved in n-hexane at room temperature (5 mL of n-hexane per 1 mmol of NPs). Then, the NPs were precipitated, again by the addition of warm ethanol (1 mL of n-hexane and 1 mL of ethanol). The final material was obtained following centrifugation at 9000 rpm for 3 min. The properties of C1-C3 samples were compared with the properties of β-core NaYF_4_:18%Yb^3+^, 2%Er^3+^ material prepared according to the procedure outlined above without the presence of SA (WSAC); the only difference of synthesis was a change of the composition of organic solutions—1 mmol of NPs was obtained in 10 mL of OA and 10 mL of 1-ODE, and this material was obtained after thermal treatment at 300 °C for 55 min. For the synthesis of β-core@β-shell UCNPs without the presence of SA, the α-shell NaYF_4_ material was also prepared according to the procedure outline above, without the presence of SA after thermal treatment at 200 °C for 55 min.

#### 2.2.4. Synthesis of β-core@β-shell UCNPs

The nominal (assumed) ratio of NPs:OA:1-ODE:SA for the synthesis of β-core@β-shell UCNPs was the same as that in the procedure described in Section 2.2.2. Synthesis of Nanomaterials.

A portion of the hot solution with α-shell NaYF_4_ NPs (the mixture of solvents ODE, OA, and SA with dispersed α-shell NaYF_4_ NPs), the temperature of which was approximately 70 °C, was placed directly in the flask with the solution of β-core NaYF_4_: 18%Yb^3+^, 2%Er^3+^ NPs under the flow of nitrogen. It was used that 1 mL of the solution with α-shell NPs was added to 1 mL of the solution with β-core NPs. Then, the mixture of α-shell and β-core NPs was outgassed under a high vacuum (<10^−1^ mbar) from 70 °C to 100 °C and outgassed at this temperature for 30 min. After the outgassing, the solution was heated to 300 °C under the flow of nitrogen. The solution was kept at this temperature for 35 (CS1 sample), 45 (CS2 sample), or 55 min (CS3 sample) and then cooled to 70 °C under the flow of nitrogen.

The procedure of UCNPs separation was identical to the procedure described in Section 2.2.3. Synthesis of β-core NaYF_4_:18%Yb^3+^, 2%Er^3+^ and α-shell NaYF_4_ NPs. The hot solution with UCNPs (its temperature was ~70 °C) was poured into the centrifuge probes with pure hot ethanol (its temperature was ~70 °C) and the mixture of solutions was centrifuged at 9000 rpm for 3 min. The precipitation of the UCNPs from the mixture of organic solutions was obtained by the addition of hot ethanol (applied at the volume ratio of 1 mL of supernatant: 1 mL of ethanol). After the centrifugation, the solution was removed, and the precipitate of NPs was dissolved in n-hexane at room temperature (it was assumed that 1 mmol of NPs was dissolved in 5 mL of n-hexane). The UCNPs were precipitated by the addition of hot ethanol (the assumed volume ratio was 1 mL of n-hexane to 1 mL of ethanol). The pure UCNPs were obtained following centrifugation at 9000 rpm for 3 min.

The properties of CS1-CS3 samples were compared with the properties of β-core NaYF_4_: 18%Yb^3+^, 2%Er^3+^@β-core NaYF_4_ material prepared according to the above procedure without the presence of SA (WSACS); the only difference of synthesis of this material was based on the change of the composition of organic solutions—1 mmol of NPs was obtained by the mixture of 10 mL of OA and 10 mL of 1-ODE after thermal treatment at 300 °C for 55 min.

### 2.3. Characterization of Materials

The obtained materials were characterized using several analytical methods. X-ray diffraction (XRD) measurements were carried out on a Bruker AXS D8 Advance diffractometer (Billerica, MA, USA) with Cu *K*_α_ radiation (*λ* = 0.154 nm) and a step size of 0.05° in the wide-angle range (10–80°). The XRD patterns of the prepared UCNPs were measured for fresh powder samples, and the obtained X-ray diffraction patterns were compared with those reported in the Crystallography Open Database (COD). The Zetasizer Nano ZS Malvern device (Malvern, Worcestershire, United Kingdom) was applied to analyze the size of NP aggregates in n-hexane solutions. All measurements using this equipment were carried out at 25 °C for 1 mL of solutions with NPs in a concentration close to 1 mg∙mL^−1^. For the transmission electron microscopy (TEM) measurements, the NPs were deposited in the form of their n-hexane colloid solutions on copper grids covered with a holey carbon film and transferred to a JEOL 1400 (C1-C2 samples) or Hitachi HT7700 (C3 and CS1-CS3 samples) electron microscope operating at 80–120 kV. The NPs were dispersed in n-hexane directly after their synthesis, and their solution was deposited on copper grids with carbon film before the measurements using a TEM. The ATR-FTIR spectra were recorded using the Vertex 70 Bruker FTIR spectrophotometer (Billerica, MA, USA, resolution 4 cm^−1^, number of scans = 64, in the range 4000–400 cm^−1^). The studied samples were put into the Platinum ATR diamond F vacuum A225/Q equipment, which was connected to the spectrophotometer. The FTIR spectra were recorded for the samples at room temperature (RT) under atmospheric pressure. The spectrum without any sample (“background spectrum”) was scanned and subtracted from all of the recorded spectra. The photoluminescence properties of core@shell UCNPs in the form of n-hexane colloid solutions (5 mg∙mL^−1^) were studied at room temperature using a PIXIS:256E Digital CCD Camera equipped with an SP-2156 Imaging Spectrograph from Princeton Instruments (Sarasota, FL, USA) and QuantaMaster^TM^ 40 spectrophotometer from Photon Technology International (Birmingham, NY, USA), equipped with an R928 photomultiplier from Hamamatsu (Hamamatsu, Japan). A continuous multiwavelength, 2 W CW diode laser (CNI) was used as the excitation source at 975 nm, coupled to a 200 µm optical fiber and collimator (from Thorlabs, Newton, NJ, USA). All of the PL spectra were corrected for the spectral response of the equipment. The beam size and laser powers were determined by a 10A-PPS power meter from Ophir Photonics (Darmstadt, Germany).

## 3. Results and Discussion

### 3.1. Structure and Morphology of NPs

**Figure 1:**
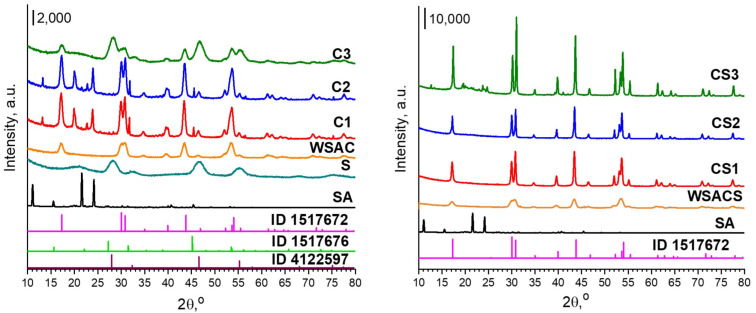
XRD patterns performed for α-shell (S), β-core (C1-C3), β-core material obtained without stearic acid (WSAC), core@shell materials (CS1-CS3), core@shell material obtained without stearic acid (WSACS), and pure stearic acid (SA). The obtained XRD patterns were compared from the XRD pattern typical for the crystal structure of sodium-yttrium fluoride (NaYF_4_) in hexagonal coordination (Crystallography Open Database (COD) ID 1517672) and cubic coordination (Crystallography Open Database (COD) ID 4122597) and sodium-triyttrium fluoride (NaY_3_F_10_) in cubic coordination (Crystallography Open Database (COD) ID 1517676).

The crystallographic structures of the nanoparticles were determined by XRD (Figure 1). According to the results, the core@shell NPs were obtained in the hexagonal crystal phase, which is typical of NaREF_4_ materials synthesized by the reaction in high boiling point solvents at high temperature (approximately 300 °C). The number and the positions of the reflections in the XRD patterns of the nanomaterials obtained are in strong agreement with the XRD pattern from the Crystallography Open Database (COD ID 1517672). The XRD patterns of sample CS3 show reflections that are assigned to the crystal phase of pure stearic acid, which indicates that the time of synthesis of the core@shell UCNPs had an impact on the deposition of stearic acid on the surface of the nanoparticles. The reflections at the same 2θ also appeared in the XRD patterns recorded for samples C1 and C2. Several weak reflections in the XRD patterns recorded for samples C1 and C2 can be assigned to the cubic phase of sodium-triyttrium fluoride (NaY_3_F_10_). As indicated in Figure 1, the deposition of stearic acid on the surface of core materials can lead to the stabilization of β-phase form, but the longer the synthesis of core@shell NPs, the more likely it is to lead to the deposition of a layer of stearic acid on the external surface and the formation of the core@shell NPs coordinated by organic ligands.

**Figure 2:**
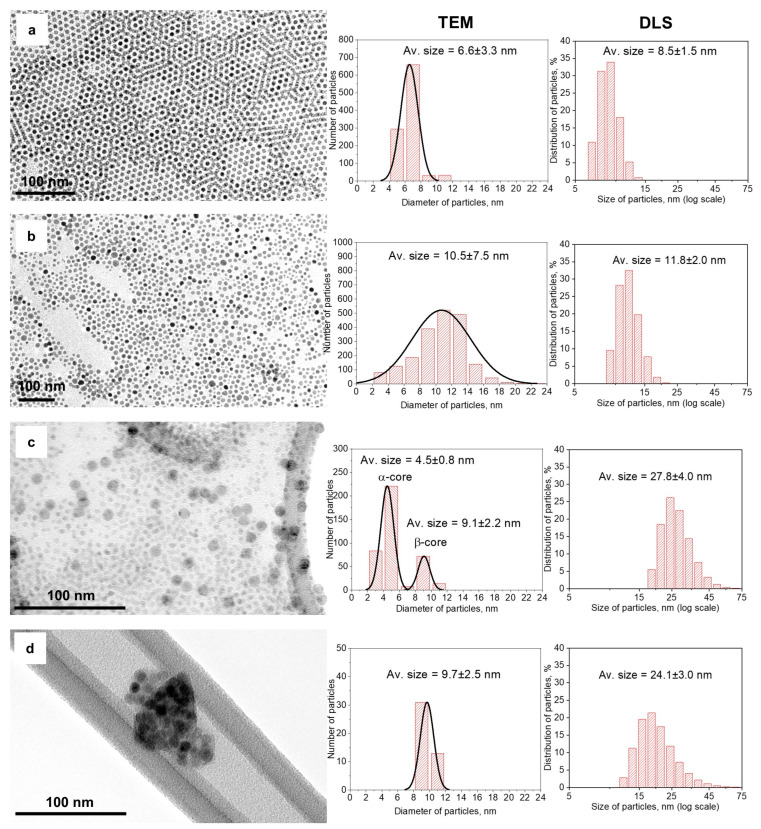
TEM images recorded for β-NaYF_4_: 18%Yb^3+^, 2%Er^3+^ NPs obtained at 300 °C: (**a**) C1 sample (for 35 min), (**b**) C2 sample (for 45 min), (**c**) C3 sample (for 55 min), and (**d**) WSAC and their particle size distribution estimated using TEM and DLS techniques.

Figure 2 presents TEM images of the obtained β-NaYF_4_: 18%Yb^3+^, 2%Er^3+^ NPs. Depending on the reaction conditions, the NPs’ average sizes were between 4.5 and 10.5 nm. Interestingly, the short-time synthesis at 300 °C (for 35 min) resulted in the homogenous NPs with an average size of 6.6 ± 3.3 nm, which formed an ordered hexagonal arrangement (Figure 2a, C1 sample). This phenomenon was not observed for the colloid solutions of NPs obtained at 300 °C after 45 min (Figure 2b, C2 sample). It suggests that the stearic acid can act as a reagent, stabilizing the size of NPs and influencing how NPs are deposited. The presence of stearic acid on the surface of C1 NPs induced a different ordering of the particle layer. The distance between NPs visible in Figure 2a is approximately 1 nm, corresponding to a monolayer of stearic acid molecules between NPs. The presence of stearic acid induced the development of a densely packed hexagonal layer (Figure 2a). It has been reported in [25,26] that this kind of packing can appear when NPs interact with the solvent molecules with certain specific dielectric properties. Solvents with high dielectric constants (e.g., the dielectric constant of stearic acid is 2.3) and a high melting point (e.g., 69 °C in the case of stearic acid) are known to reduce packing densities and enable the formation of “quasi-crystalline” structures. The hexagonal packing of the layer in the mixture of stearic acid and oleic acid indicates strong attraction between neighboring NaYF4: 18%Yb^3+^, 2%Er^3+^ NPs or between the NPs and stearic acid [25,27]. The strength of the lateral interactions depends on the solvent, and in the case of NP solutions with SA, they may include nonadditive collective particle interactions that contribute to the formation of quasi-crystalline superlattices, similar to a hexagonal honeycomb.

**Figure 3:**
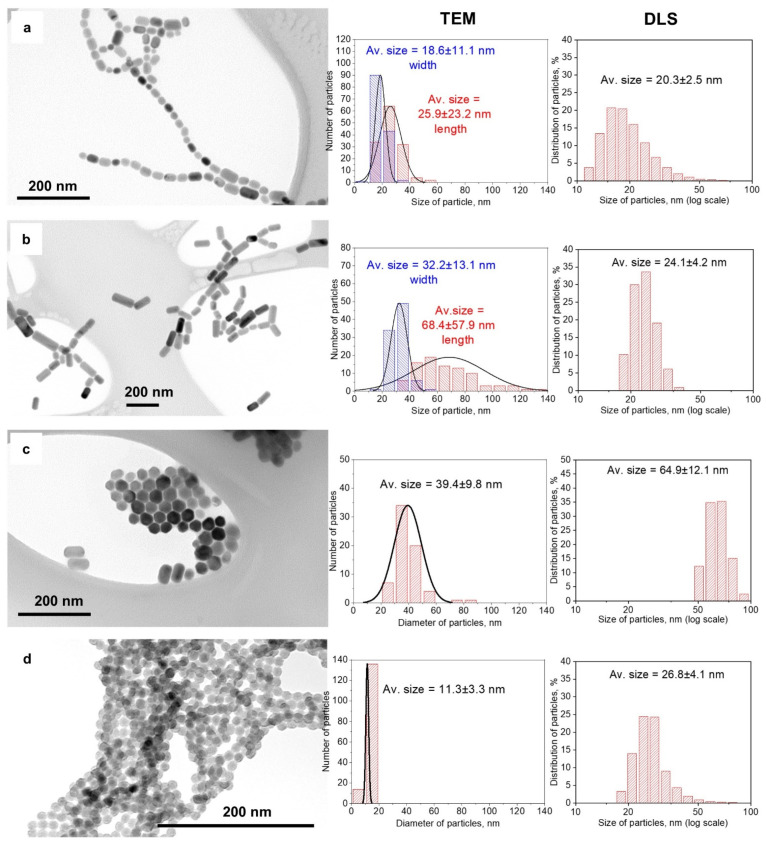
TEM images recorded for β-NaYF_4_: 18%Yb^3+^, 2%Er^3+^@β-NaYF_4_ NPs obtained at 300 °C: (**a**) CS1, (**b**) CS2, (**c**) CS3, and (**d**) WSACS samples and their particle size distribution estimated using TEM and DLS technique.

TEM images recorded for β-core@β-shell NPs (Figure 3) show that the synthesis time can play a crucial role in the formation of NPs with different morphologies. The core@shell NPs occurred in the form of small rods with an average size in the range of 18.6–68.4 nm. It was shown that the CS3 NPs were characterized by hexagonal morphology (Figure 3c). The NPs obtained in the short-time synthesis had the shapes of nanorods (Figure 3a,b, CS1 and CS2 samples). After the long-time synthesis, the separation of NPs was complicated (Figure 3c). It can be explained by the agglomeration of NPs during a long time of thermal synthesis and the precipitation of β-core@β-shell NPs with the layer of stearic acid on their external surface.

The synthesis of NPs without stearic acid (WASC and WASCS samples) allows spherical particles to be obtained whose average size is similar in the case of both β-core materials (the average size of C3 and WASC NPs is 9.1 ± 2.2 nm and 9.7 ± 2.5 nm, respectively). The differences are seen in the case of core@shell materials. The NPs prepared without stearic acid (WASCS sample) are much smaller than the analogous CS3 NPs because the comparison of the size of nanoparticles using TEM images shows the slight growth of NPs in the case of WASCS—the average size of these NPs is 11.3 ± 3.3 nm. The average size of NPs in the case of the CS3 sample is 39.4 ± 9.8 nm. This difference can suggest that the presence of SA molecules during the synthesis of core@shell UCNPs can accelerate the growth of nanocrystals. The comparison of TEM images recorded for CS3 and WASCS samples shows that the addition of SA to the mixture of organic solutions positively affects the crystallization of nanoparticles to the hexagonal phase.

Additionally, the obtained NPs were analyzed by the ICP-OES technique to confirm whether the composition agreed with the theoretical value. The results are collected in Table 2 and are similar to the theoretical values, which assume a 1:1 core to shell ratio and concentration of dopant ions of 1% in the case of Er^3+^ and 9% in the case of Yb^3+^ ions.

The nanorod form of the obtained UCNPs indicates that the crystallization of NPs is based on the deposition of reagents (dissolved α-shell NPs) during the synthesis, mainly on the external surface of β-core NPs. The obtained results suggest that the long-time synthesis can lead to the polymerization of SA and the agglomeration of NPs. The use of stearic acid can also lead to the obtaining of NPs with the hexagonal structure (Figure 3c).

**Figure 4:**
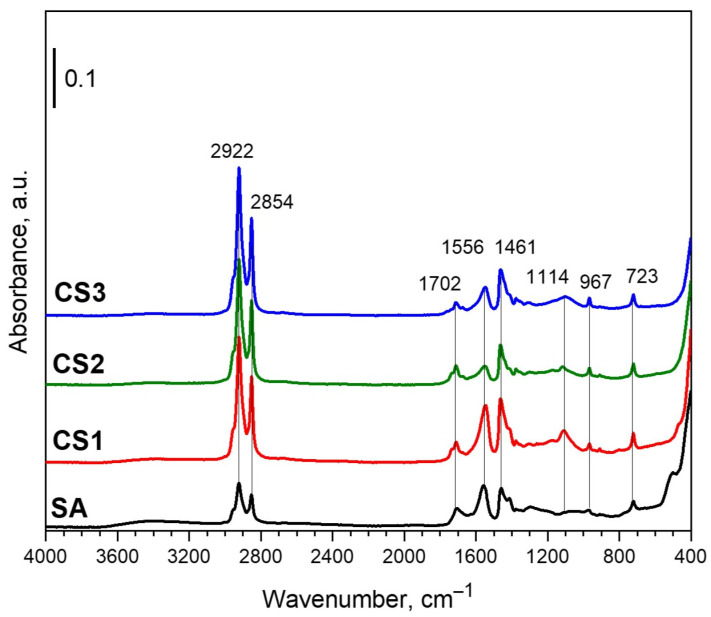
FITR spectra recorded for core@shell NPs (CS1, CS2, and CS3 samples) obtained at 300 °C and pure stearic acid (SA).

FTIR spectra were recorded for core@shell materials and compared with the FTIR spectrum of pure stearic acid (SA) (Figure 4) to confirm the presence of SA on the surface of the obtained NPs. The high-frequency region’s absorption bands at approximately 2922 and 2854 cm^−1^ are assigned to the asymmetric and symmetric stretching vibrations of −CH_2_− bonds, respectively [28,29]. In the low-frequency region, the band at 1702 cm^−^^1^ is attributed to the vibrations of the −COOH group from stearic acid (SA). The FTIR spectra’ adsorption bands at 1461 and 1556 cm^−1^ correspond to the asymmetric and symmetric stretches of the −COO− group [30]. The band at approximately 967 cm^−1^ and 723 cm^−1^ can be assigned to the –COH out-of-plane deformation and the rocking vibrations of the –CH_2_− bond, respectively [31,32]. A comparison of the FTIR spectra recorded for the core@shell samples and pure SA shows that all of the samples are covered with a layer of stearic acid. This means that all of the samples contain core@shell NPs with the additional shell layer on the external surface of the NPs. The samples are in the form of inorganic β-core@inorganic β-shell@organic amorphous shell (β-NaYF_4_: 18Yb^3+^, 2%Er^3+^@β-NaYF_4_@SA). The presence of the SA phase on the external surface of NPs is promising for their medical application, e.g., it was shown in [33] that a nanosystem based on dextran (Dex), stearic acid (SA), and poly ethyl glycol (PEG) was used for encapsulation of anti-viral drug zidovudine (AZT). SA and PEG components provided stability to the nanosystem and aided in controlled drug release. Additionally, as a potential drug delivery system, SA/Dex NPs exhibited superior quality compared to bare Dex NPs in terms of stability, drug release, and nanoparticle uptake by cells.

Table 3 presents the selected parameters of synthesis (volume ratio of organic solutions, time, and temperature of reaction) and the main size of NPs prepared using the other thermal methods, taken from literature, and the corresponding results obtained in this study [19,34,35,36].

The comparison of the morphology properties of NPs prepared using selected RE source and organic solvents shows that the addition of SA as an organic solvent and the application of RE chlorides can lead to the obtaining of good-sized core@shell NPs (below 50 nm) with a structure similar to the nanorods (in the case of shorter synthesis) or a hexagonal shape (in the case of more prolonged synthesis).

### 3.2. Luminescent Properties of NPs

The emission spectra, decay times, and dependences of their emission intensity on the laser power density were measured to investigate the spectroscopic properties of the core@shell structures obtained in and without the presence of SA.

**Figure 5:**
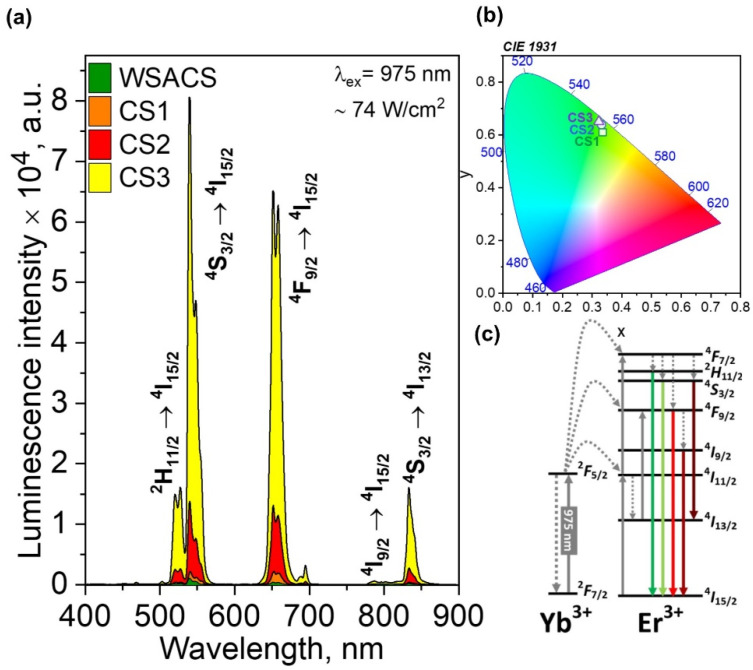
The emission spectra (**a**), CIE chromaticity diagrams (**b**), and proposed UC mechanisms (**c**) responsible for the observed emission for core@shell structures: CS1, CS2, CS3, and WSACS. Emission spectra were measured upon excitation with the 975 nm wavelength of the colloids in hexane (1 mg∙mL^−1^).

In all of the obtained samples, Er^3+^ may undergo five characteristic transitions upon excitation with 975 nm laser radiation (Figure 5a), from which the one ^4^S_3/2_ → ^4^I_15/2_ gives the most intense emission band assigned to the present core@shells. The observed spectroscopic properties are also reflected in the green color of the emission visible to the naked eye. The chromaticity diagram confirms the observed emission color (Figure 5b). The highest emission intensity characterizes the CS3 sample among all of the samples studied. The emission of the CS3 sample was almost five times higher than that of the next most emitting sample. It is worth noting that the bands at the emission spectrum recorded for the WSACS sample are characterized by a much smaller intensity than the analogous CS3 sample.

The most critical factor responsible for the lower emission intensity of NPs obtained without the presence of SA is their smaller size in comparison to the remaining NPs. Moreover, the differences in shell thickness might be responsible for the observed emission properties. From the TEM images, the shell thickness can be estimated to be approximately 12, 22, 35, and 2 nm for CS1-CS3 and WSACS NPs, respectively. The larger NPs are, the thicker the shell is and the better the luminescence intensity under 975 nm excitation.

The most important conclusion derived from the obtained results is that the preparation of NPs in the presence of SA leads to larger NPs with better emission intensity. To obtain NPs in OA/OD system, the reaction time must be longer than the applied 55 min. Therefore, the addition of SA allows for a shortening of the reaction time.

**Figure 6:**
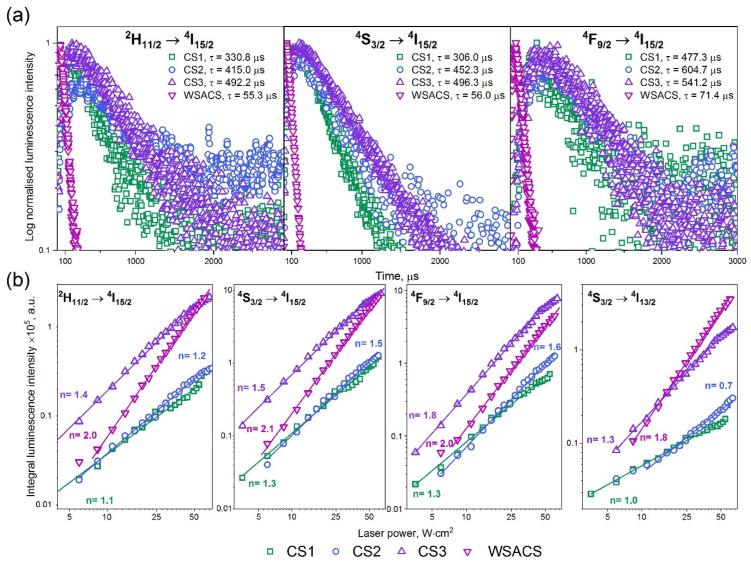
The decay time (**a**) and double-logarithmic plots of the upconversion emission intensity versus laser power density (**b**) of core@shell structures CS1 (green square), CS2 (blue circle), CS3 (violet triangle) and WSACS (inverted purple triangle) upon 975 laser excitation ((**a**) pulse and (**b**) continuous excitation). All of the presented data was collected for NPs in the form of hexane colloids (5 mg∙mL^−1^).

Based on the luminescence decays measured for the three main transitions (^2^H_11/2_ → ^4^I_15/2_, ^4^S_3/2_ → ^4^I_15/2_, ^4^F_9/2_ → ^4^I_15/2_), the luminescence lifetimes were calculated and collected in Figure 6a. The obtained samples exhibited relatively long emission decays [37]. NPs received without SA showed the shortest decays, which means they are not as well protected from solvent molecules as NPs with SA on their surface. Moreover, the small size of WSACS NPs makes them prone to quenching.

To confirm the mechanism of the observed upconversion luminescence for the obtained structures, the dependences of their emission intensity on the laser power density were measured (Figure 6b). The number of photons involved for excitation of each energy level was calculated from the obtained results. For this purpose, the equation describing the relation between the UC intensity *I* and the pumping excitation power density, *P*, was used:

(1) $$I∼P^{n}$$

where *n* is the number of photons required to populate the excited state (*n* > 0.5) [38].

Yb^3+^ ions act as sensitizers in the presented system, absorbing the excitation radiation and transferring energy to Er^3+^ ions. Under irradiation with a 975 nm laser, the excitation of Yb^3+^ ions from ^2^F_7/2_ to ^2^F_5/2_ excited state occurs, followed by energy transfer to the Er^3+^ ions [4]. Subsequently, absorption of the next photon yields Er^3+^ ions in their ^2^H_11/2_ and ^4^S_3/2_ excited states after non-radiative relaxation from the ^4^F_7/2_ excited state. Another possible excitation pathway yields Er^3+^ ions in their ^4^F_9/2_ excited state after absorption of the second photon and energy transfer from Yb^3+^ to the ^4^I_13/2_ excited state of Er^3+^ ions.

Theoretically, to excite the ^2^H_11/2_, ^4^S_3/2_, ^4^F_9/2_ energy levels, sequential absorption of two photons is needed (Figure 5c). For the core@shell structures CS1, CS2, CS3, the calculated number of photons is between 1 and 2 for each measured transition, which is lower than the theoretical value. However, if the experimentally determined slope coefficient exceeds 1, it can be assumed that two-photon excitation of a given energy level occurred. The low values of the slope coefficients indicate the presence of quenching processes, competing with radiative relaxations from the Er^3+^ excited states.

## 4. Conclusions

The synthesis of UCNPs with the core@shell structure by precipitation and Ostwald ripening in the OA/OD/SA system is an effective method for preparing materials based on NaYF_4_ doped with Yb^3+^ and Er^3+^ ions (β-NaYF_4_: 18%Yb^3+^, 2%Er^3+^@β-NaYF_4_). The TEM images and XRD patterns of NPs doped with Yb^3+^ and Er^3+^ ions confirmed that the proposed synthesis method could be applied to synthesize core@shell structures. Depending on the synthesis parameters, the final nanoparticles were characterized by a unique shape (with hexagonal coordination) whose size is below 50 nm for β-NaYF_4_: 18%Yb^3+^, 2%Er^3+^@β-NaYF_4_ core@shell UCNPs.

The use of SA as an additive to the synthesis allowed the synthesis of larger NPs compared to analogous ones obtained without the presence of SA. As the obtained NPs were of larger sizes, their luminescence was also more intense. From the results acquired, it can be assessed that the presence of SA promotes the faster growth of NPs; thus, SA affects the kinetics of the precipitation reaction. Interestingly, it was found that the presence of SA molecules on the surface of core NPs obtained in the shortest reaction time used influences the ordering of NPs. NPs have been observed to self-organize into hexagonal structures. Moreover, the measured luminescence properties, and especially luminescence decays, showed that NPs with SA on their surface are quenched by water molecules to a lesser extent than NPs prepared in the presence of OA and OD only. The obtained results can be significant in the case of upscaling the production of NPs, where parameters such as synthesis time and reaction kinetics are often of crucial importance.

## Figures and Tables

**Table 1:** The list of obtained and characterized materials.

No.	Sample Name	The Volume Ratio of Organic Solutions	Conditions of Synthesis
1.	C1	OA:SA:ODE = 1:1:2	β-core—35 min at 300 °C
2.	C2	OA:SA:ODE = 1:1:2	β-core—45 min at 300 °C
3.	C3	OA:SA:ODE = 1:1:2	β-core—55 min at 300 °C
4.	WSAC	OA:ODE = 1:1	β-core—55 min at 300 °C
5.	CS1	OA:SA:ODE = 1:1:2	β-core—35 min at 300 °C α-shell—35 min at 200 °C β-core@β-shell—35 min at 300 °C
6.	CS2	OA:SA:ODE = 1:1:2	β-core—45 min at 300 °C α-shell—45 min at 200 °C β-core@β-shell—45 min at 300 °C
7.	CS3	OA:SA:ODE = 1:1:2	β-core—55 min at 300 °C α-shell—55 min at 200 °C β-core@β-shell—55 min at 300 °C
8.	WSACS	OA:ODE = 1:1	β-core—55 min at 300 °C α-shell—55 min at 200 °C β-core@β-shell—55 min at 300 °C

**Table 2:** The concentration of lanthanide ions in the obtained NPs.

Sample Name	Er at. %	Yb at. %	Y at. %
CS1	1.13 ± 0.10	8.81 ± 0.29	90.06 ± 3.93
CS2	1.32 ± 0.60	8.48 ± 0.58	90.20 ± 3.39
CS3	1.01 ± 0.31	7.30 ± 0.43	91.69 ± 2.96
WSACS	1.02 ± 0.05	9.20 ± 0.46	89.78 ± 5.27

**Table 3:** Comparison of the example synthesis conditions of core@shell nanoparticles doped with lanthanide ions.

Material	RE Source	Organic Mixture (Volume Ratio)	Time of Synthesis, min	The Temperature of Synthesis, °C	The Size of core@shell Nanoparticles, nm ^a^	Morphology	Ref.
NaGdF_4_:Nd^3^^+^, Yb^3^^+^ @CaF_2_:Eu^3^^+^	acetates	OA/ODE (2:3)	30 (α-shell) 90 (core@shell) 120—total time	150 (α-shell) 300 (core@shell)	150–159	hexagonal particles	[34]
β-NaYF_4_: 18%Yb^3+^, 2%Er^3+^ @β-NaYF_4_	oleates	OA/ODE (1:6) OA/ODE (2:5) OA/ODE (1:1) OA/ODE (3:2) OA/ODE (3:1)	60 (β-core) 60 (core@shell) 120—total time	300 (β-core) 290 (core@shell)	48.9 ± 2.2 (l), 26.9 ± 2.2 (w) 28.6 ± 1.9 (l), 23.7 ± 1.9 (w) 33.3 ± 1.9 (l), 30.1 ± 1.4 (w) 27.6 ± 1.7 (l), 25.3 ± 1.4 (w) 36.2 ± 1.7 (l), 32.4 ± 2.2 (w)	nanorods	[19]
β-NaGdF_4_: Yb/Er @β-NaYF_4_	TFA	OA/ODE/OM (1:2:1) (β-core) OA/ODE (1:1) (β-core and core@shell)	15 (β-shell) 30 (β-core) 60 (core@shell) 105—total time	310 (β-shell) 310 (β-core) 290 (core@shell)	16.5	hexagonal particles	[35]
NaLaF_4_:Nd^3+^ @NaLaF_4_	acetates	OA/ODE (3:7)	30 (α-shell) 60 (β-core) 60 (core@shell) 210—total time	170 (α-shell) 310 (β-core) 310 (core@shell)	20 ± 3 (l), 80 ± 12 (w)	nanorods	[36]
CS1	chlorides	OA/ODE/SA (1:2:1)	35 (α-shell/β-core) 35 (core@shell) 70—total time	200 (α-shell) 300 (β-core) 300 (core@shell)	26.6 ± 7.8	nanorods	this work
CS2	chlorides	OA/ODE/SA (1:2:1)	45 (α-shell/β-core) 45 (core@shell) 90—total time	200 (α-shell) 300 (β-core) 300 (core@shell)	70.1 ± 25.5	nanorods	this work
CS3	chlorides	OA/ODE/SA (1:2:1)	55 (α-shell/β-core) 55 (core@shell) 110—total time	200 (α-shell) 300 (β-core) 300 (core@shell)	39.4 ± 9.8	hexagonal particles	this work

TFA—trifluoroacetates, OA—oleic acid, ODE—1-octadecene, OM—oleylamine, SA—stearic acid, (l)—length, (w)—width, ^a^—from TEM images.

## Data Availability

The data presented in this study are available on request from the authors. Data is contained within the article.
